# Burden of asthma by severity and exacerbation frequency among adult patients naive to biologic asthma therapy: A Finnish cohort study

**DOI:** 10.1016/j.jacig.2025.100453

**Published:** 2025-03-14

**Authors:** Hannu Kankaanranta, Arja Viinanen, Anton Klåvus, Mariann I. Lassenius, Helga Haugom Olsen, Kaisa Nieminen, Annina Lyly, Paula Kauppi, Lauri Lehtimäki

**Affiliations:** a Faculty of Medicine and Health Technology, Tampere University, Tampere, Finland; b Department of Respiratory Medicine, Seinäjoki Central Hospital, Seinäjoki, Finland; c Department of Internal Medicine and Clinical Nutrition, Krefting Research Centre, Institute of Medicine, University of Gothenburg, Gothenburg, Sweden; d Department of Pulmonary Diseases, Turku University Hospital, Turku, Finland; e Department of Pulmonary Diseases and Clinical Allergology, University of Turku, Turku, Finland; f Medaffcon Oy, Espoo, Finland; g Medical Department, BioPharmaceuticals, AstraZeneca, Oslo, Norway; h Medical Department, BioPharmaceuticals, AstraZeneca, Espoo, Finland; i Skin and Allergy Department, Helsinki University Hospital and University of Helsinki, Helsinki, Finland; j AstraZeneca, Espoo, Finland; k Department of Pulmonary Medicine, Heart and Lung Center, Helsinki University Hospital and University of Helsinki, Helsinki, Finland; l Allergy Centre, Tampere University Hospital, Tampere, Finland

**Keywords:** Asthma, burden of disease, exacerbations, mortality, comorbidities, health care resource utilization, sick leaves, disability pensions, treatment

## Abstract

**Background:**

Understanding the disease burden and characteristics of asthmatic patients with frequent exacerbations is important for optimal disease management and outcomes. Asthma, and especially severe uncontrolled asthma, associates with an increased disease burden, but the comparison across asthma severity and exacerbation frequency is largely missing.

**Objective:**

We sought to assess the association of asthma severity and exacerbation frequency with medication use, mortality, sick leaves, disability pensions, health care contacts, and comorbidities among Finnish patients with asthma.

**Methods:**

National longitudinal retrospective data on adult patients naive to biologic asthma therapy were used to match patients on the basis of age, sex, and region across 4 subgroups (5525 patients in each) of nonsevere or severe asthma with infrequent or frequent exacerbations. The clinical characteristics, mortality rates, and morbidity across the subgroups were analyzed.

**Results:**

Exacerbation frequency associated with an increased disease burden regardless of asthma severity. Comorbidities, health care contacts, sick leaves, and disability pensions cumulated in patients with frequent exacerbations, peaking with severe asthma. In patients with severe asthma and frequent exacerbations, the all-cause mortality rate ratio was 1.9-fold (*P* < .001) versus patients with nonsevere asthma and infrequent exacerbations. Patients with frequent exacerbations were also exposed to high cumulative corticosteroid doses.

**Conclusions:**

Despite improved outcomes in asthma over the past decades, a substantial proportion of patients experience frequent exacerbations. These patients are multimorbid and at increased risk of mortality. Exacerbation frequency, rather than asthma severity, seems to be the main factor associated with an increased disease burden. Clinical awareness should be raised to improve the management and outcomes for these patients.

Asthma remains a public health issue, affecting approximately 200,000 to 400,000 (4%-8%) people of the Finnish population of 5.6 million.^1^^,^^2^ Optimizing asthma management has been a target of public health interventions, leading to improvements in disease control and decreases in health care resource utilization and costs related to productivity loss.^3^, ^4^, ^5^ Nevertheless, a proportion of patients remain inadequately controlled despite optimized inhalation therapy. Furthermore, severe asthma is often managed in primary health care, without appropriate assessment by an asthma specialist.^1^^,^^6^^,^^7^

Biologic therapies became available to patients with severe uncontrolled asthma through national reimbursement in Finland in 2020. Access is restricted to patients on optimal high-dose inhaled corticosteroid (ICS)-containing combination treatment with 3 or more exacerbations per year or 2 exacerbations per year despite oral corticosteroid (OCS) use. However, patients not fulfilling these criteria may experience a high disease burden.

Insights into the disease burden of asthmatic patients with frequent exacerbations are important to ensure optimal disease management and outcomes. Many studies have described the comorbidities in the overall asthma population and in the subpopulation of severe asthma.^8^, ^9^, ^10^ Also, it has been documented that asthma, particularly in the presence of other comorbidities, is associated with an increased risk of sick leaves and disability pensions when compared with age-matched people without asthma.^11^ However, the comparison of disease burden across asthma severity and exacerbation frequency is missing.

We used longitudinal, nationwide data to investigate the disease burden among patients with severe and nonsevere asthma stratified by exacerbation frequency to provide a holistic overview of how asthma severity and exacerbation frequency affect medication use, mortality, sick leaves, disability pensions, health care contacts, and comorbidities in patients not on biologic asthma therapy. Our data complement previous studies, providing novel insights into the link between asthma severity, exacerbations, and disease burden.

## Methods

### Data and permission

This was a retrospective registry-based study including nationwide data on diagnoses, primary and secondary health care contacts, drug purchases, causes of deaths, long-term sick leaves (10th day onward), and disability pensions with related diagnoses from 4 different registers (National Institute for Health and Welfare, Social Insurance Institution, Statistics Finland, and Centre for Pensions). Data were linked by a unique personal identification number by the central permission authority, Findata (permission no. THL/2385/14.02.00/2021), in accordance with the Act on the Secondary Use of Health and Social Data in Finland.^12^

### Cohort formation

**Fig 1:**
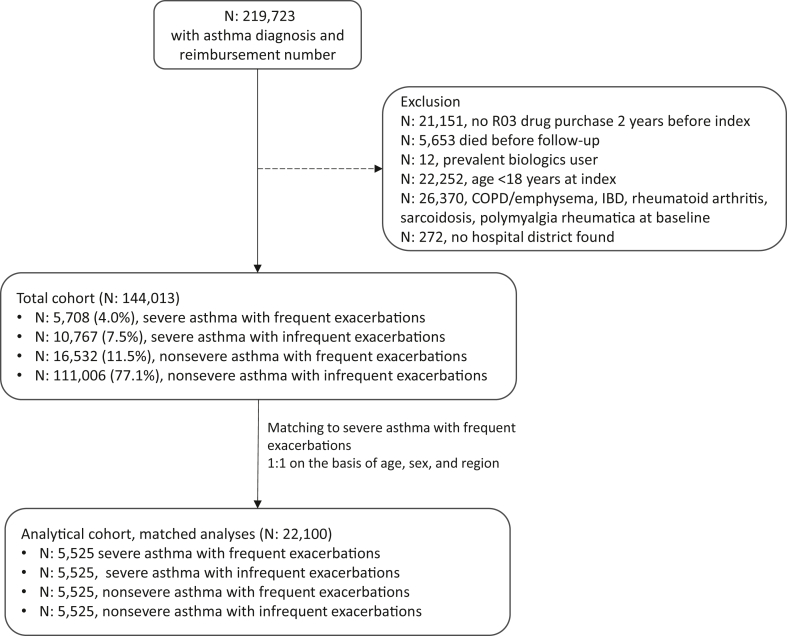
Cohort formation and exclusion criteria applied for the total asthma cohort and the matched cohort (1:1 matching with age, sex, and region to severe asthma with frequent exacerbations). *IBD*, Inflammatory bowel disease.

Adult patients with an asthma diagnosis (*International Classification of Diseases, Tenth Revision* [*ICD-10*] code J45∗/J46∗) who had made at least 1 drug purchase for obstructive airway diseases (R03∗ Anatomical Therapeutic Class) and had received reimbursement from the national drug reimbursement system for asthma during the period 2015 to 2016 were included in the raw cohort (N = 219,723). See this article’s Online Repository at www.jaci-global.org for a description of the reimbursement criteria for chronic asthma. Patients using biologics for asthma (n = 12) were excluded as were patients with other conditions requiring corticosteroids (n = 26,370) (excluded conditions: *ICD-10* diagnosis inflammatory bowel disease, rheumatoid arthritis, sarcoidosis, or polymyalgia rheumatica). In addition, patients with a diagnosis of chronic obstructive pulmonary disease (COPD) between 5 years before and at the index date (n = 19,278) were excluded to reduce confounding. This left a total cohort of 144,013 patients (Fig 1). New COPD diagnoses during follow-up were censored (observed in 3%-8% of subgroups). Baseline comorbidities were assessed 5 years before index.

### Asthma severity and exacerbation status

The total cohort of 144,013 patients was split into 4 mutually exclusive subgroups: nonsevere asthma with infrequent exacerbations, nonsevere asthma with frequent exacerbations, severe asthma with infrequent exacerbations, and severe asthma with frequent exacerbations (Fig 1; see also Table E1 in this article’s Online Repository at www.jaci-global.org).

Severe asthma was defined in line with the European Respiratory Society/American Thoracic Society guidelines,^13^ requiring a daily fluticasone propionate (FP) dose greater than or equal to 800 μg/d or equivalent (allowing 80% adherence to an FP dose of 1000 μg/d) and at least 1 other controller (see Table I) per sliding window of 4 consecutive purchases during the 2-year baseline. Patients not fulfilling this criterion were considered to have nonsevere asthma.

**Table I:** Clinical characteristics of the patients stratified by asthma severity and exacerbation frequency

Characteristics	Nonsevere asthma		Severe asthma		Overall *P* value	Significant *post hoc* tests
	Infrequent exacerbations (n = 5525)	Frequent exacerbations (n = 5525)	Infrequent exacerbations (n = 5525)	Frequent exacerbations (n = 5525)		
Age (y)	63 (51, 74)	63 (51, 74)	63 (51, 74)	63 (51, 74)	>.9	
Sex						
Female	3891 (70)	3891 (70)	3891 (70)	3891 (70)	>.9	
Male	1634 (30)	1634 (30)	1634 (30)	1634 (30)	>.9	
Years from first reimbursement	9 (4, 15)	9 (4, 15)	9 (4, 16)	14 (6, 22)	<.001	NSFE/SFE NSIE/SFE NSIE/SIE SFE/SIE
Age (y) at first reimbursement∗	54 (41, 64)	53 (41, 63)	52 (41, 62)	47 (35, 58)	<.001	All
Charlson comorbidity index†	1 (0, 1)	1 (1, 1)	1 (0, 1)	1 (1, 1)	<.001	All
Mean daily ICS dose, FP equivalent (μg)‡§	246 (137, 410)	308 (185, 451)	831 (657, 985)	862 (657, 1005)	<.001	All
No ICS purchases	195 (3.5)	114 (2.1)	0 (0)	0 (0)		
Mean daily OCS dose, prednisolone equivalent (mg)‡§	0.82 (0.41, 0.82)	1.85 (1.64, 3.28)	0.82 (0.68, 0.82)	2.46 (1.64, 3.97)	<.001	NSFE/NSIE NSFE/SFE NSIE/SFE NSFE/SIE SFE/SIE
No OCS purchases†	4450 (80.5)	296 (5.4)	3593 (65)	242 (4.4)		
No. of add-on maintenance drug classes used (LTRA/LABA/LAMA)‡					<.001	All
0	2221 (40)	1252 (23)	0 (0)	0 (0)		
1	2492 (45)	2503 (45)	2862 (52)	1684 (30)		
2	752 (14)	1463 (26)	2234 (40)	2585 (47)		
3	60 (1.1)	307 (5.6)	429 (7.8)	1256 (23)		
High SABA use, >450 actuations/12 mo	656 (12)	1214 (22)	1409 (26)	2277 (41)	<.001	All
Respiratory antibiotic use‡‖	1497 (27)	3010 (54)	2141 (39)	3796 (69)	<.001	All

Data are presented as n (%) or median (25th quartile, 75th quartile).

*LABA*, Long-acting β-agonist; *LAMA*, long-acting muscarinic antagonist; *LTRA*, leukotriene receptor antagonist; *NSFE*, nonsevere asthma and frequent exacerbation; *NSIE*, nonsevere asthma and infrequent exacerbation; *SFE*, severe asthma and frequent exacerbation; *SIE*, severe asthma and infrequent exacerbation.

∗ The time point of when the patient was granted reimbursement for asthma the first time, used to indicate asthma onset.

† 5 y before index.

‡ During the 2-y baseline.

§ Among patients with purchases.

‖ At least 1 purchase of doxycycline, amoxicillin, amoxicillin clavulanate, azithromycin, or clarithromycin.

Exacerbation status was determined on the basis of health care contacts and OCS purchases. Frequent exacerbators were required to have at least 2 emergency room visits or 1 hospitalization during the 2-year baseline, with asthma (J45.x) as primary diagnosis (or secondary diagnosis if the primary diagnosis was a respiratory infection [J0.x-J22.x]) or acute asthma (J46.x) as primary or secondary diagnosis. Patients who purchased more than 600 mg prednisolone equivalent (>3 bursts of prednisolone 40 mg/d for 5 days) per rolling 365 days during the 2-year baseline were also considered frequent exacerbators. Patients not fulfilling these criteria were considered to have infrequent exacerbations.

High short-acting β_2_-agonist (SABA) use was defined as more than 450 actuations (irrespective of dose) per 12 months.^14^

### Matching

From the total cohort of 144,013 patients, those with severe asthma and frequent exacerbations (n = 5,708 [4% of the total cohort]) were used as reference and the other subgroups were matched 1:1 on the basis of hospital district, age, and sex to this subgroup (Fig 1). A match was found for 5,525 patients with severe asthma and frequent exacerbations, resulting in a final matched cohort with 22,100 patients. The matched subgroups were followed from January 1, 2017, to December 31, 2020, or till death. All analyses were conducted and reported in these age-, sex-, and hospital district–matched subgroups.

### Mortality

Annual mortality rates were calculated by dividing the number of deaths by the total follow-up time in the subgroup. COPD was used as a censoring event. Main causes of death were used to define respiratory (*ICD-10* J∗) and cardiovascular (*ICD-10* I∗) mortality. Mortality rates were compared between groups using Poisson regression.

### Health care resource utilization

Primary and specialized care contacts during follow-up were assessed and reported as contacts per patient year (PPY). Bootstrapping (10,000 samples) was used for 95% CI estimation. Corresponding estimates stratified by subgroup were computed.

### Sick leaves and disability pensions

The mean cumulative number of long-term sick leave days (10th day onward) and disability pension–related days were assessed in patients of working age (<65 years). Patients were censored from the analysis on their 65th birthday.

### OCS, ICS, and SABA use

The mean cumulative values of OCS, ICS, and SABA use during baseline and follow-up were assessed using mean cumulative function models. The delta change between annual point estimates of the mean cumulative function corresponds to average annual use.

### Statistical analysis

Continuous and nominal variables, such as demographic characteristics, clinical characteristics, and comorbidities, were described using standard statistical measures (ie, number of observations/missing observations, mean, SD, median, and first and third quartile). All categorical variables were summarized with absolute and relative frequencies.

Differences between populations were tested using the Kruskal-Wallis test, followed by the Dunn test for continuous variables, chi-square test for categorical variables when all the expected counts for the cells of the contingency table were 5 or higher, and the Fisher exact test for categorical variables when any of the expected counts for the cells of the contingency table were lower than 5. For pairwise comparisons between groups, *P* values were adjusted using the Holm method for controlling family-wise error rates. *P* values less than .05 were considered statistically significant. All data processing and statistical analysis were performed using R (version 4.0.3; Vienna, Austria).^15^

## Results

### Patient cohorts

In the total asthma cohort of 144,013 patients, at index, most patients had nonsevere asthma and infrequent exacerbations (77.1%), followed by nonsevere asthma and frequent exacerbations (11.5%) and severe asthma in combination with infrequent exacerbations (7.5%) and with frequent exacerbations (4.0%). The proportion of patients with severe and frequently exacerbating asthma remained stable during follow-up. Patients were matched on the basis of age, sex, and region using the patients with severe and frequently exacerbating asthma as the reference group. The matching was successful for 96.8% of patients with severe asthma and frequent exacerbations, resulting in 5525 patients in each of the 4 subgroups of patients with severe/nonsevere and frequent/infrequent exacerbations (Fig 1). Results are reported for the matched subgroups.

At index, the median age was 63 years (25th, 75th percentile: 51, 74) and 70% of patients were female. Patients with severe and frequently exacerbating asthma had the youngest median age at asthma medication reimbursement onset of 47 years (25th, 75th percentile: 35, 58), on average 5 years before the other groups (Table I). Patients with severe and frequently exacerbating asthma at index used a mean dose of 862 μg of FP equivalent ICS per day in addition to on average 2.46 mg of prednisolone equivalent OCS per day, and 70% had used at least 2 additional controllers (leukotriene receptor antagonist/long-acting β-agonist/long-acting muscarinic antagonist). Moreover, 41% of the severe frequent exacerbators used more than 450 SABA actuations per year at index. The use of respiratory antibiotics ranged from 27% in patients with nonsevere asthma and infrequent exacerbations to 54% in those with nonsevere asthma and frequent exacerbations, and from 39% in patients with severe asthma and infrequent exacerbations to 69% in those with severe asthma and frequent exacerbations (Table I).

During follow-up, exacerbation rates and ICS, OCS, and SABA classification showed only slight changes from index (see Tables E2 and E3 in this article’s Online Repository at www.jaci-global.org). In the subgroups, the drug doses (per patient) cumulated during the 4-year follow-up period were as follows: OCS, 458, 2,384, 814 mg, and 3,028 mg; ICS FP equivalent, 416,000, 505,000, 1,100,000, and 1,164,000 μg; and SABA actuations, 518, 739, 859, and 1,201 in the subgroups of nonsevere asthma and infrequent exacerbations, nonsevere asthma and frequent exacerbations, severe asthma and infrequent exacerbations, and severe asthma and frequent exacerbations (Table E3).

### Disease burden

Disease burden captured mortality, long-term sick leaves, disability pensions, health care contacts, and comorbidities.

#### Mortality

**Fig 2:**
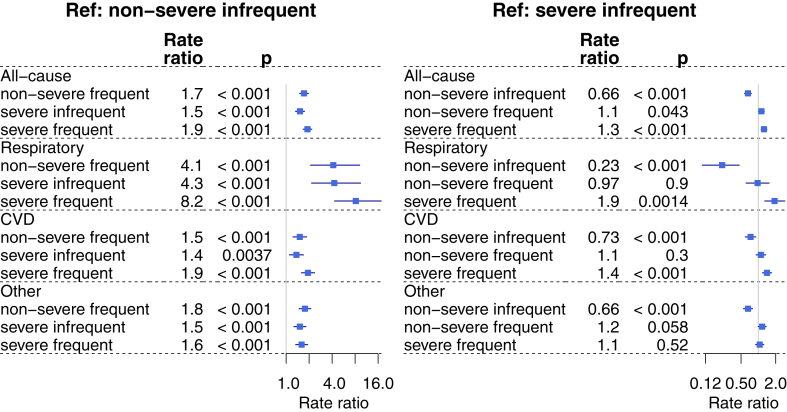
Forest plot of mortality rate ratio in comparison with nonsevere asthma and infrequent exacerbations (*left*) and severe asthma and infrequent exacerbations (*right*), stratified by all-cause, respiratory, and cardiovascular mortality. *CVD*, Cardiovascular disease.

Overall, 1923 deaths were observed during the 4-year follow-up, increasing with exacerbation frequency and asthma severity (number of deaths: 326, 550, 468, and 579 in groups with nonsevere asthma and infrequent exacerbations, nonsevere asthma and frequent exacerbations, severe asthma and infrequent exacerbations, and severe asthma and frequent exacerbations, respectively). Annual mortality rates were the highest in patients with severe asthma and frequent exacerbations (on average 0.029 deaths PPY), followed by patients with nonsevere asthma and frequent exacerbations (0.027 deaths PPY), severe asthma and infrequent exacerbations (0.023 deaths PPY), and nonsevere asthma with infrequent exacerbations (0.016 deaths PPY) (see Fig E1 in this article’s Online Repository at www.jaci-global.org). Compared with the subgroup with nonsevere asthma and infrequent exacerbations, mortality was significantly higher in all other subgroups (rate ratio, 1.5-1.9; *P* < .01 for all). The increased mortality was observed for respiratory causes (rate ratio, 4.1-8.2; *P* < .001), cardiovascular causes (rate ratio, 1.4-1.9; *P* < .01), and other causes (rate ratio, 1.5-1-8; *P* < .001) (Fig 2).

Compared with patients with severe asthma and infrequent exacerbations, all-cause mortality was higher in patients with frequent exacerbations (both nonsevere and severe) (rate ratio, 1.1-1.3; *P* < .05) (Fig 2).

#### Sick leaves and disability pensions

Sick leave and disability pension analyses were limited to the working-age population (<65 years) of 2964 patients (54%) per subgroup and assessed as working days lost due to sick leaves lasting at least 10 days or being on disability pension. During the 4-year follow-up, sick leaves were observed in 32.7%, 41.2%, 36.9%, and 44.0% of patients with nonsevere asthma and infrequent exacerbations, nonsevere asthma and frequent exacerbations, severe asthma and infrequent exacerbations, and severe asthma and frequent exacerbations, respectively. The corresponding proportions of patients with disability pension–related days during follow-up were 14.1%, 21.0%, 19.2%, and 22.9% in the subgroups.

**Fig 3:**
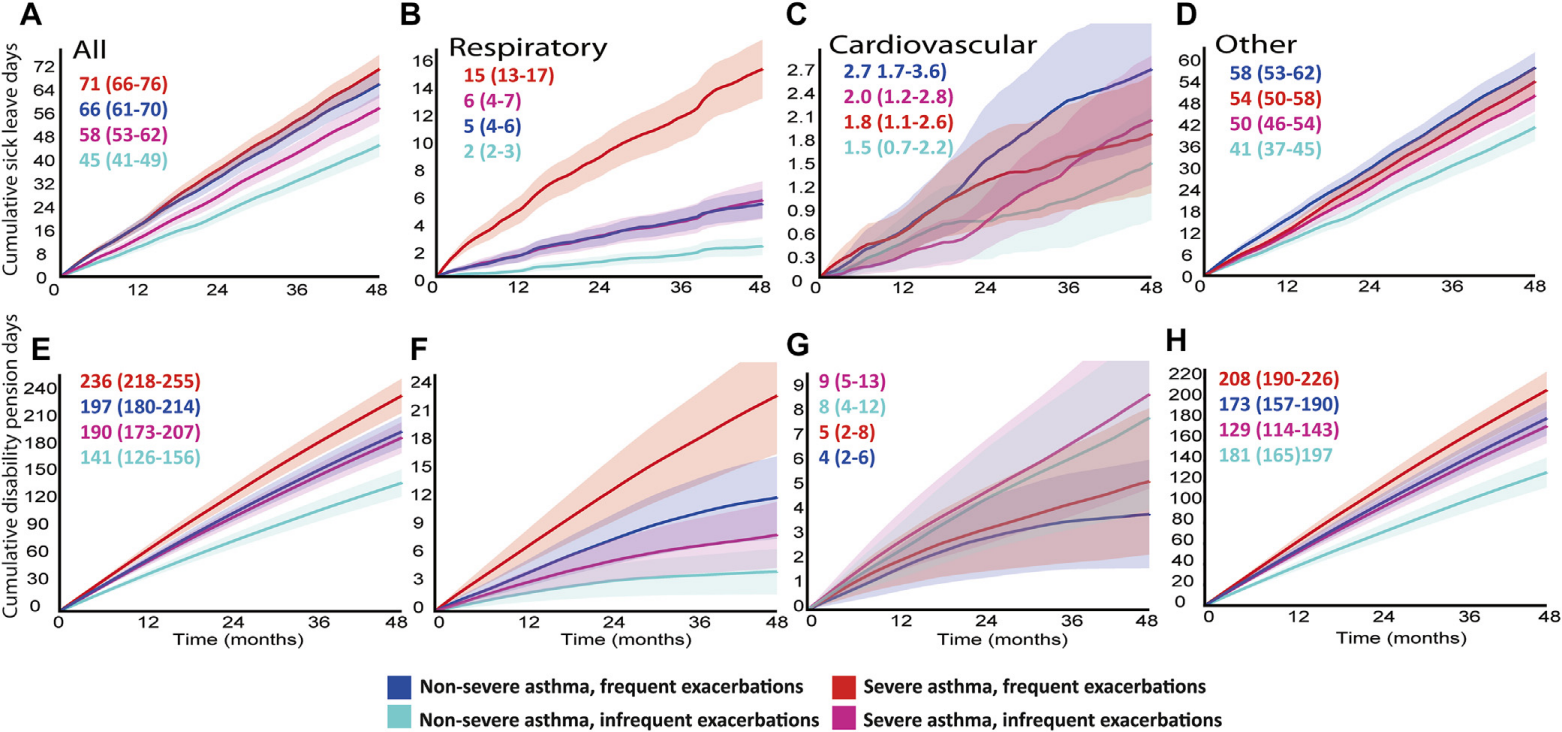
**A-H,** Mean (95% CI) cumulative number of absenteeism (Fig 3, *A-D*) and disability-related days per patient (Fig 3, *E-H*) during follow-up in patients younger than 65 years (All: all-cause; other: unrelated to respiratory and cardiovascular causes). The 2964 working-age patients with severe asthma and frequent exacerbations had 71 absenteeism days (18 days annually) and 236 disability days (59 days annually) on average per patient during follow-up.

The mean cumulative number of all-cause long-term sick leave days as well as disability pension–related days during follow-up was the highest in patients with severe asthma and frequent exacerbations, followed by those with nonsevere asthma and frequent exacerbations and with severe asthma and infrequent exacerbations (Fig 3). The same was true for respiratory-related sick leave and disability pension days, whereas the difference in cardiovascular-related sick leave days overlapped between the groups (Fig 3). Diagnoses related to sick leaves and disability pensions are provided in Tables E4 and E5 (in the Online Repository available at www.jaci-global.org).

#### Health care contacts

**Fig 4:**
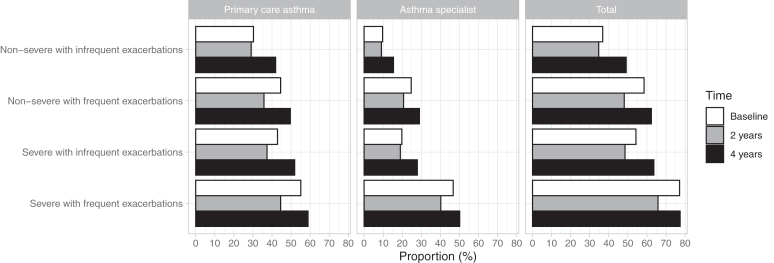
Proportion of patients with visits to primary health care for asthma and asthma specialists during baseline and follow-up (2 and 4 years) in subgroups.

The increased morbidity burden observed in patients with frequent exacerbations was reflected in health care contacts during follow-up (see Fig E2 in this article’s Online Repository at www.jaci-global.org). The number of all health care–related visits (primary and secondary care contacts) was the highest in subjects with severe asthma and frequent exacerbations, with an average of 50 (95% CI, 46-53) contacts PPY, mainly driven by primary care (18 [95% CI, 18-19] contacts PPY), home care (25 [95% CI, 22-29] contacts PPY), and secondary (specialty) care (5 [95% CI, 5-5] contacts PPY) outpatient visits, compared with subjects with nonsevere and infrequent exacerbations with 32 (95% CI, 29-35) visits of any type of contact PPY. Across all subgroups, respiratory-related contacts represented a small fraction of all contacts. Asthma visits (to both primary care and asthma specialists) increased by disease severity and exacerbation frequency. During the 4 years of follow-up, 77% of patients with severe asthma and frequent exacerbations had an asthma-related visit, 59% had a primary care visit for asthma, and 50% had visited an asthma specialist (Fig 4).

#### Comorbidity burden

The differences between groups in mortality and health care use were not directly related to respiratory causes, suggesting that the increased burden of frequently exacerbating asthma might relate to comorbidities. To evaluate this, the presence of comorbidities was analyzed. At baseline, the median (interquartile range [IQR]) number of unique diagnoses, on an *ICD-10* 3-character level, were 6 (IQR, 3-10), 8 (IQR, 5-13), 7 (IQR, 3-10), and 10 (IQR, 5-15) in the subgroups of nonsevere asthma and infrequent exacerbations, nonsevere asthma and frequent exacerbations, severe asthma and infrequent exacerbations, and severe asthma and frequent exacerbations, respectively (*P* < .001 across all subgroups and any subgroup comparison). For a large number of diseases, prevalence differed across subgroups (Table II; see also Table E6 in this article’s Online Repository at www.jaci-global.org). The most frequent diagnoses with at least 5% prevalence in any of the 4 asthma subgroups were respiratory (n = 10), cardiovascular (n = 5), metabolic (n = 4), dental (n = 4), musculoskeletal (n = 13), and other (n = 16) diagnoses (Table E6).

**Table II:** Comorbidities (%) of unique *ICD-10* codes on a 3-character level, with a prevalence of ≥5% in any subgroup, on the basis of data from 5 y preceding the index∗

Diagnosis		Nonsevere asthma		Severe asthma		Overall *P* value	Significant *post hoc* tests	Fold difference	
		Infrequent exacerbations (n = 5525)	Frequent exacerbations (n =5525)	Infrequent exacerbations (n =5525)	Frequent exacerbations (n = 5525)			NSFE vs NSIE	SFE vs NSIE
Respiratory	J20: Acute bronchitis	13.0	**27.0**	19.0	**35.0**	<.001	All	2.1	2.6
	J06: Acute upper respiratory infections of multiple and unspecified sites	17.0	**28.0**	**22.0**	**32.0**	<.001	All	1.6	1.8
	J12-J18: Pneumonia	6.6	19.0	12.0	**29.0**	<.001	All	2.9	4.5
	J01: Acute sinusitis	12.0	**20.0**	15.0	**26.0**	<.001	All	1.7	2.2
	J32: Chronic sinusitis	2.7	7.6	4.5	11.0	<.001	All	2.8	4.1
	J30: Vasomotor and allergic rhinitis	4.1	6.6	6.0	7.8	<.001	NSFE/NSIE SIE/NSIE SFE/NSIE SFE/NSFE SFE/SIE	1.6	1.9
	J33: Nasal polyp	1.7	6.4	1.9	6.2	<.001	NSFE/NSIE SFE/NSIE SIE/NSFE SFE/SIE	3.8	3.7
	J31: Chronic rhinitis, nasopharyngitis, and pharyngitis	2.2	3.5	3.6	6.1	<.001	NSFE/NSIE SIE/NSIE SFE/NSIE SFE/NSFE SFE/SIE	1.6	2.8
	J47: Bronchiectasis	0.5	1.4	1.8	6.1	<.001	NSFE/NSIE SIE/NSIE SFE/NSIE SFE/NSFE SFE/SIE	2.6	11.6
	J22: Unspecified acute lower respiratory infection	1.5	3.6	2.6	5.9	<.001	All	2.4	4.0
CVD	I10: Essential (primary) hypertension	**31.0**	**36.0**	**33.0**	**38.0**	<.001	All	1.2	1.2
	I48: Atrial fibrillation and flutter	8.9	11.0	9.4	13.0	<.001	NSFE/NSIE SFE/NSIE SIE/NSFE SFE/NSFE SFE/SIE	1.3	1.4
	I25: Chronic ischemic heart disease	7.8	9.5	8.6	11.0	<.001	NSFE/NSIE SFE/NSIE SFE/NSFE SFE/SIE	1.2	1.4
	I50: Heart failure	4.0	7.6	6.2	10.0	<.001	All	1.9	2.5
	I49: Other cardiac arrhythmias	4.9	5.9	5.6	6.3	.015	NSFE/NSIE SFE/NSIE	1.2	1.3

*CVD*, Cardiovascular disease; *NSFE*, nonsevere asthma and frequent exacerbation; *NSIE*, nonsevere asthma and infrequent exacerbation; *SFE*, severe asthma and frequent exacerbation; *SIE*, severe asthma and infrequent exacerbations.

∗ Heatmap proportions stratified by respiratory and CVD diagnoses. The complete table including metabolic, dental, musculoskeletal, and other diagnoses is found in the Online Repository (bolded prevalence, ≥20%).

Compared with the nonsevere infrequent exacerbators, the highest difference in prevalence was observed for bronchiectasis in those with severe and frequently exacerbating asthma (0.5% vs 6.1% [11.5-fold higher]), followed by pneumonia (6.6% vs 29.0%), chronic sinusitis (2.7% vs 11%), and acute lower respiratory tract infections (1.5% vs 5.9%). In general, respiratory and cardiovascular diagnoses were most frequent in those with frequent exacerbations, peaking with severe asthma, even if the differences were more modest compared with patients with nonsevere asthma and infrequent exacerbations (1.2- to 2.5-fold higher) (Table II). Across other diagnoses, the same increase in prevalence by exacerbation frequency was observed (Table E6).

## Discussion

This study indicates that, irrespective of asthma severity, frequent exacerbations are associated with a higher disease burden in terms of comorbidities, mortality, health care resource utilization, sick leaves, disability pensions, and cumulative SABA and OCS use.

Asthma control status and resource use have improved over the past decades in Finland, because of national asthma and allergy programs with targeted population-based interventions and education during the periods 1994 to 2004 and 2008 to 2018.^3^^,^^4^ Further developments within pharmacogenetics and treatment responses may allow personalized treatments.^16^ In addition, more targeted treatments have become available to treat patients with severe asthma who continue to exacerbate while on high-dose inhaled therapy. Despite this progress, the prevalence of severe, uncontrolled asthma has remained relatively stable during recent years. As evident from our study, during the years 2017 to 2020 a substantial proportion of patients still experienced frequent exacerbations while on inhaled therapy. In fact, of the total asthma cohort, 15.5% had frequent exacerbations despite receiving on average a moderate-high ICS dose. Considering the flat ICS dose–response in asthma, these patients (ie, both those on low-moderate and high-dose ICS) may be considered to represent “difficult-to-treat” asthma, albeit it cannot be excluded that some patients in the nonsevere group were undertreated.^17^

In the analyses matching patients with severe asthma and frequent exacerbations to the other subgroups, frequent exacerbations were linked to high comorbidity burden, high OCS use, frequent respiratory antibiotic purchases (54%-70% of patients), increased mortality, and increased health care resource utilization, demonstrating a significant disease burden in these patients. SABA use was also high, with 41% of the severe frequent exacerbators and 22% of the nonsevere frequent exacerbators using SABA at more than 450 actuations per year. Globally, SABA overuse is common and is associated with increased asthma exacerbations and mortality.^18^^,^^19^

In patients with severe asthma and frequent exacerbations, average cumulative OCS use reached 3000 mg at 4 years of follow-up. This is of concern, considering that the use of 1 to 2 short courses of OCS is associated with an increased risk of adverse events,^20^^,^^21^ and asthma guidelines recommend minimizing OCS use to reduce the cumulative burden of adverse effects.^22^^,^^23^

Asthma, per se, associates with an increased mortality rate.^24^ The mortality rate of 0.029 PPY (29/1000 patient year) in patients with severe and frequently exacerbating asthma is higher than that observed in other studies, ranging from 5-9/1000 patient years in asthma to 12-25/1000 patient years in severe asthma.^25^ These estimates have not considered exacerbation frequency and the patients have been on average almost 10 years younger compared with those in our study. Little to no data exist on patients with nonsevere asthma and frequent exacerbations, with a mortality rate of 0.027 (27/1000 patient years) in this study, almost as high as in corresponding patients with severe asthma. Notably, when comparing patients with frequent exacerbations (nonsevere/severe asthma) with patients with nonsevere asthma and infrequent exacerbations, not only respiratory mortality was increased, but also mortality due to cardiovascular and other causes.

Existing literature supports the role of high doses of corticosteroids affecting comorbidities such as metabolic conditions, cardiovascular disease, and hypertension.^9^^,^^26^ However, emerging evidence suggests that asthma and exacerbation-related systemic inflammation associate with extrapulmonary conditions such as depression, cardiovascular disease, and metabolic diseases.^27^^,^^28^ Thus, both inflammation and corticosteroid-related adverse events could be the potential link between exacerbations and the increased disease burden and mortality evidenced in this study.

Encouraging results of treating inflammation in other conditions come from anti–TNF-α drugs, where the treatment of one inflammatory condition may alleviate the other.^29^ For example, anti–TNF-α drugs in inflammatory bowel disease have improved not only the condition but also coexisting mood disorders.^30^ Whether biologic therapies would result in similar multiorgan benefits in the asthma population remains a topic for future studies.

National treatment guidelines for asthma recommend annual follow-up visits in primary health care for patients using continuous asthma medication.^23^ Patients with persistent symptoms and exacerbations should be referred to a specialist for diagnostic and phenotypic assessment and for consideration of add-on therapies.^22^^,^^31^ In contrast to the recommendation, our study shows that primary health care contacts as well as specialist visits remain low across the subgroups. Although patients with severe asthma and frequent exacerbations had on average 18 primary health care visits annually, 93% of all visits were unrelated to respiratory causes. Asthma-related primary health care visits were observed in 45% at 2 years and in 59% at 4 years, and only 50% had a visit to an asthma specialist during the 4-year follow-up. Furthermore, in the subgroup of patients with nonsevere asthma and frequent exacerbations, appropriate treatment escalation was not implemented because the mean ICS dose remained low-moderate during follow-up. Acknowledging that data on treatment adherence were missing, overall, our findings suggest that compliance with clinical treatment guidelines may still be suboptimal, to the detriment of these patients at risk.

The strength of this study comes from the matched subgroup setting including both nonsevere and severe asthmatic patients as well as those with frequent and infrequent exacerbations, giving a full overview of the current asthma population in Finland. This allows for novel investigation of the relationship between asthma severity and exacerbation frequency and its effect on the burden of disease. Even if subgroups were matched for age, sex, and region, some biases may persist regarding sociodemographic and patient factors affecting the outcomes (eg, smoking history). Some of the included patients may also have used private health care providers. Such data were not included in the analyses because private health care is not captured in the national registries used for the study. Hence, the comorbidity burden and health care contacts we present are conservative estimates.

Further limitations come from the nature of register data and not having information on asthma-related symptoms, thus having to rely on medication use as well as health care contacts to determine severity and exacerbation status. Several exacerbations may go unnoticed if they were not related to a purchase of OCSs and undertreatment or overtreatment in relation to asthma exacerbations and severity could not be assessed. Asthma is associated with an increased risk for respiratory infections and thus we can assume that including only long-term sick leaves likely underestimated the number of sick leave days.

Despite initiatives to optimize asthma management, a substantial proportion of patients naive to biologic asthma therapy experience frequent exacerbations and high corticosteroid and SABA use. These patients are multimorbid and at increased risk of mortality. Exacerbation frequency, rather than asthma severity per se, appears to be the main factor associated with an increased disease burden, even if severe asthma augments the findings. Thus, exacerbations should be a key factor when evaluating patients, directing more attention to reasons for exacerbations, adherence to treatment, patient inflammatory phenotype, and the optimization of care pathways and asthma management. Furthermore, our study highlights the need for a paradigm change wherein difficult-to-treat asthma is viewed as a multimorbid condition benefiting from a holistic approach, with the assessment and treatment of other morbidities. Hopefully in the future, this would translate into improved outcomes.^28^

## Disclosure statement

Funding for the conduct of this study was received by AstraZeneca.

Disclosure of potential conflict of interest: A. Viinanen, L. Lehtimäki, P. Kauppi, and H. Kankaanranta have received scientific committee consultancy fees from AstraZeneca for the conduct of the study. A. Viinanen reports fees for consultation or lectures from Airsonett, AstraZeneca, Boehringer Ingelheim, Chiesi, and GlaxoSmithKline. L. Lehtimäki reports fees for consultations, lectures, or clinical trials from ALK, AstraZeneca, Berlin Chemie, Boehringer Ingelheim, Chiesi, GlaxoSmithKline, Menarini, Mundipharma, Novartis, Orion, and Sanofi. P. Kauppi reports lecturing fees for Sanofi and research fees from Theravance, outside the submitted work; and is the Head of the Finnish Respiratory Society. H. Kankaanranta reports fees for consultations or lectures from AstraZeneca, Boehringer Ingelheim, Chiesi, Covis Pharma, GlaxoSmithKline, Merck Sharp & Dohme (MSD), Novartis, Orion Pharma, and Sanofi-Genzyme, outside the submitted work; is an asthma and allergy research professor funded by the Hermann Krefting Foundation; and his work is supported by ALF agreement (grant from the Swedish state under the agreement between the Swedish government and the county councils), the Swedish Research Council, the Asthma and Allergy Research Foundation (Sweden), the Tampere Tuberculosis Foundation (Tampere, Finland), and The Competitive State Research Financing of the Expert Responsibility Area of Tampere University Hospital (Tampere, Finland). The employer of A. Klåvus and M. I. Lassenius, Medaffcon Oy, has received funding for the conduct of the study. A. Lyly is currently employed at Helsinki University Hospital and conducted this research when employed at AstraZeneca. H. H. Olsen and K. Nieminen are employees of AstraZeneca and may have stock ownership and/or stock options or interests in the company.
