# Supplementary material for: Burden of asthma by severity and exacerbation frequency among adult patients naive to biologic asthma therapy: A Finnish cohort study
Source: J Allergy Clin Immunol Glob. 2025 Mar 14;4(2):100453. doi: 10.1016/j.jacig.2025.100453 (PMC12018094; doi:10.1016/j.jacig.2025.100453)
Supplement: Supplementary Material [file mmc1.docx]

**Burden of asthma by severity and exacerbation frequency among adult patients naïve to biologic asthma therapy: a Finnish cohort study.**

Hannu Kankaanranta^1^ (MD, PhD), Arja Viinanen^2^ (MD, PhD), Anton Klåvus^3^ (MSc Tech.), Mariann I. Lassenius^3^ (PhD), Helga Haugom Olsen^4^ (MD, PhD), Kaisa Nieminen^5^ (PhD), Annina Lyly^6^ (MD, PhD), Paula Kauppi^7^ (MD, PhD), and Lauri Lehtimäki^8^ (MD, PhD)

**Supplementary data**

**Supplementary methods**

The Social Insurance in Finland covers the reimbursement of drugs that are linked with certain medical conditions such as asthma in this study with the reimbursement number 203. The entitlement to medicine reimbursement at a special rate is granted to asthma patients fulfilling the below criteria, and the application is filed by the treating physician.

The 203 right to special reimbursement for chronic asthma is granted when regular medication to reduce bronchial inflammation has lasted for at least 6 months and is continuing.

In addition to asthma symptoms, the diagnosis should be based on one of the following findings:

- In spirometry and bronchodilation assay, forced vital capacity (FVC) or forced expiratory volume in one second (FEV1) is improved by at least 12% and 200 ml, respectively.
- In a two-week PEF monitoring
  - a bronchodilation response of at least three times at least 15 % and, in patients aged 12 years and over, an additional at least 60 l/min, or
  - diurnal variation (between morning and evening values measured before the opening medicine) at least three times at least 20 % and, in patients aged 12 years and over, at least 60 l/min.
- In a treatment trial containing a corticosteroid
  - FEV1 improves by at least 15% and 200 ml or
  - the mean PEF value (compared to 3-5 days before and after treatment) improves by at least 20% and by at least 60 l/min in patients aged 12 years and over.
- In methacholine challenge test (patients 12 years of age and older), moderate to severe contractile sensitivity (PD20FEV1 ≤ 0.6 mg).
- In exercise challenge test, EVH (eucapnic voluntary hyperventilation) test (in patients aged 10 years and older) or in exposure to mannitol, FEV1 is reduced by at least 15%.

In order to receive a reimbursement decision for asthma, the statement written by a physician when applying for the reimbursement for the patient, must describe the results of the lung function tests and a spirometry printout and/or PEF monitoring must be attached. In addition to information on the disease, the statement must also include a treatment plan in accordance with good medical practice.

**Supplementary Figure 1. Annual mortality rates, and average mortality rate during follow-up in subgroups**

**Supplementary Figure 2. Number of healthcare contacts per patient year of subgroups stratified by contact type. Outpatient – specialty care outpatient visit; outpatient phc – primary healthcare outpatient visit; home care – primary healthcare given at home; PPY- per patient-year.**

Supplementary Table 1. Age and sex distribution of the unmatched subgroups

|  | Non-severe with infrequent exacerbations  N = 111,006 | Non-severe with frequent exacerbations  N = 16,532 | Severe with infrequent exacerbations  N = 10,767 | Severe with frequent exacerbations  N = 5,708 | Overall p-value | Significant post-hoc tests |
| --- | --- | --- | --- | --- | --- | --- |
| Age median (25^th^, 75^th^ quartile) | 57 (41,69) | 63 (49,74) | 62 (49, 72) | 63 (51,74) | <0.001 | All |
| Female, N (%) | 70,407 (63%) | 11,389 (69%) | 6,841 (64%) | 3,998 (70%) | <0.001 | NSFE/NSIE SFE/NSIE SIE/NSFE SFE/SIE |
| Male, N (%) | 40,599 (37%) | 5,143 (31%) | 3,926 (36%) | 1,710 (30%) |  |  |

NSFE- non-severe asthma and frequent exacerbations; NSIE - non-severe asthma and infrequent exacerbations; SFE - severe asthma and frequent exacerbations; SIE - severe asthma and infrequent exacerbations

Supplementary Table 2. Mean number of exacerbations per patient annually during baseline and follow-up and their distribution based on OCS purchase or ER visits/hospitalisations. Events 14 or more days apart were considered separate events.

|  | **Baseline** | | **Follow-up** | | | |
| --- | --- | --- | --- | --- | --- | --- |
|  | **2015** | **2016** | **2017** | **2018** | **2019** | **2020** |
| **Non-severe with infrequent exacerbations** | **0.12** | **0.12** | **0.20** | **0.22** | **0.23** | **0.21** |
| - ER visit/Hospitalisation | 0.00 | 0.00 | 0.01 | 0.02 | 0.02 | 0.01 |
| - OCS purchase | 0.11 | 0.12 | 0.18 | 0.20 | 0.21 | 0.20 |
| **Non-severe with frequent exacerbations** | **1.47** | **1.58** | **1.10** | **1.01** | **0.99** | **0.85** |
| - ER visit/Hospitalisation | 0.11 | 0.12 | 0.06 | 0.04 | 0.04 | 0.03 |
| - OCS purchase | 1.35 | 1.46 | 1.04 | 0.97 | 0.95 | 0.82 |
| **Severe with infrequent exacerbations** | **0.23** | **0.24** | **0.38** | **0.41** | **0.39** | **0.35** |
| - ER visit/Hospitalisation | 0.01 | 0.01 | 0.03 | 0.03 | 0.03 | 0.02 |
| - OCS purchase | 0.22 | 0.22 | 0.35 | 0.37 | 0.36 | 0.33 |
| **Severe with frequent exacerbations** | **1.80** | **1.84** | **1.40** | **1.33** | **1.29** | **1.13** |
| - ER visit/Hospitalisation | 0.20 | 0.21 | 0.12 | 0.10 | 0.08 | 0.05 |
| - OCS purchase | 1.59 | 1.63 | 1.28 | 1.23 | 1.21 | 1.07 |

Supplementary Table 3 Mean annual ICS, OCS and SABA use per patient in subgroups during baseline and cumulative use during baseline and follow-up.

|  | Year | Non-severe asthma, infrequent exacerbations  (N=5,525) | Non-severe asthma, frequent exacerbations  (N=5,525) | Severe asthma, infrequent exacerbations  (N=5,525) | Severe asthma, frequent exacerbations  (N=5,525) |
| --- | --- | --- | --- | --- | --- |
| Baseline mean ICS µg FP equivalent/day | 2015 | 270 | 310 | 823 | 852 |
|  | 2016 | 272 | 322 | 838 | 897 |
| Follow-up mean ICS µg FP equivalent/day | 2017 | 277 | 335 | 788 | 835 |
|  | 2018 | 275 | 336 | 751 | 799 |
|  | 2019 | 284 | 344 | 736 | 774 |
|  | 2020 | 302 | 366 | 738 | 778 |
| **Cumulative follow-up ICS µg FP equivalent^1^** | **2017-2020** | **415,507** | **504,703** | **1,100,155** | **1,164,624** |
| Baseline mean OCS mg prednisolone equivalent/year | 2015 | 49 | 933 | 97 | 1,122 |
|  | 2016 | 51 | 1,021 | 96 | 1,164 |
| Follow-up mean OCS mg prednisolone equivalent/year | 2017 | 101 | 660 | 199 | 841 |
|  | 2018 | 122 | 607 | 215 | 783 |
|  | 2019 | 118 | 609 | 217 | 749 |
|  | 2020 | 117 | 508 | 183 | 656 |
| **Cumulative follow-up OCS mg** | **2017-2020** | **458** | **2,384** | **814** | **3,028** |
| Baseline mean SABA actuations / year | 2015 | 132 | 204 | 241 | 345 |
|  | 2016 | 131 | 205 | 230 | 349 |
| Follow-up mean SABA actuations / year | 2017 | 130 | 188 | 222 | 306 |
|  | 2018 | 126 | 182 | 220 | 303 |
|  | 2019 | 128 | 184 | 208 | 296 |
|  | 2020 | 135 | 185 | 210 | 296 |
| **Cumulative follow-up SABA actuations** | **2017-2020** | **518** | **739** | **859** | **1,201** |

^1^the cumulative ICS during follow-up is presented as the cumulative mg in four years, FP equivalent.

Supplementary Table 4. Top 15 main causes of sick leaves lasting 10 days or more, stratified by subgroup, and expressed as proportion of total sick-leave days during follow-up in prevalence order according to the severe asthma and frequent exacerbations subgroup.

| **ICD-10** | **Description** | **Non-severe asthma, infrequent exacerbations**  **(N=5,525)** | **Non-severe asthma, frequent exacerbations (N=5,525)** | **Severe asthma, infrequent exacerbations (N=5,525)** | **Severe asthma, frequent exacerbations (N=5,525)** |
| --- | --- | --- | --- | --- | --- |
| J45-J46 | Asthma | 1.5% | 3.2% | 4.0% | 13.7% |
| F32-F33 | Depression | 13.8% | 16.5% | 15.6% | 13.3% |
| M51 | Other intervertebral disc disorders | 4.0% | 2.6% | 2.3% | 3.9% |
| F41 | Other anxiety disorders | 2.1% | 2.8% | 3.1% | 3.8% |
| M54 | Dorsalgia | 3.1% | 5.3% | 4.4% | 3.8% |
| M17 | Gonarthrosis [arthrosis of knee] | 4.7% | 3.9% | 6.4% | 3.0% |
| M75 | Shoulder lesions | 5.5% | 2.1% | 2.9% | 2.4% |
| M47 | Spondylosis | 2.0% | 0.8% | 0.8% | 1.7% |
| J12-J18 | Pneumonia | 0.5% | 0.6% | 1.2% | 1.7% |
| F43 | Reaction to severe stress, and adjustment disorders | 2.0% | 1.5% | 1.8% | 1.4% |
| M19 | Other arthrosis | 1.0% | 1.6% | 1.3% | 1.2% |
| S83 | Dislocation, sprain and strain of joints and ligaments of knee | 0.6% | 0.8% | 0.7% | 1.2% |
| C50 | Malignant neoplasm of breast | 1.4% | 2.3% | 2.2% | 1.2% |
| M53 | Other dorsopathies, not elsewhere classified | 1.2% | 0.4% | 0.6% | 1.1% |
| S82 | Fracture of lower leg, including ankle | 1.5% | 0.9% | 0.9% | 1.1% |

*Causes of sick leaves lasting 10 days or more for patients younger than 65 at index were assessed. In these 2 964 (54%) patients per subgroup, sick leaves were observed in 968 (32.7%), 1220 (41.2%), 1094 (36.9%), and 1303 (44.0%) of non-severe asthma and infrequent exacerbations, non-severe asthma and frequent exacerbations, severe asthma and infrequent exacerbations, and severe asthma and frequent exacerbations respectively. For example, depression accounted for 13.3% of all sick leave days assessed in the severe asthma with frequent exacerbations subgroup.*

Supplementary Table 5. Top 15 main diagnoses of disability pensions for patients younger than 65 at index, stratified by subgroups, expressed as number of individual patients on disability pension in prevalence order according to the severe frequent exacerbator group. The percentages reflect the proportion of all patients with a disability pension period due to the respective cause in the respective subgroup.

| **ICD-10** | **Description** | **Non-severe asthma, infrequent exacerbations,** **N = 417** | **Non-severe asthma, frequent exacerbations, N = 621** | **Severe asthma, infrequent exacerbations, N = 570** | **Severe asthma, frequent exacerbations,** **N = 678** |
| --- | --- | --- | --- | --- | --- |
| F32-F33 | Depression | 21.3% | 22.2% | 22.3% | 20.5% |
| J45-J46 | Asthma | 2.9% | 4.0% | 4.4% | 12.8% |
| M51 | Other intervertebral disc disorders | 3.4% | 4.2% | 4.0% | 4.7% |
| F31 | Bipolar affective disorder | 5.5% | 2.6% | 5.3% | 3.7% |
| M17 | Gonarthrosis [arthrosis of knee] | 3.8% | 4.8% | 5.4% | 2.2% |
| F20 | Schizophrenia | 2.9% | 1.4% | 3.3% | 2.1% |
| F60 | Specific personality disorders | 1.9% | 1.4% | 3.9% | 1.8% |
| M75 | Shoulder lesions | 2.9% | 2.3% | 1.9% | 2.1% |
| M47 | Spondylosis | 2.4% | 2.6% | 1.6% | 2.2% |
| M19 | Other arthrosis | 1.4% | 2.9% | 2.1% | 1.8% |
| F41 | Other anxiety disorders | < 1.2% | 2.4% | 2.1% | 2.2% |
| F70 | Mild mental retardation | 1.9% | 1.1% | 2.3% | 1.9% |
| M54 | Dorsalgia | 1.9% | 1.3% | 2.3% | 1.6% |
| M48 | Other spondylopathies | 2.2% | 1.1% | 1.8% | 1.8% |
| M15 | Polyarthrosis | 2.2% | 1.9% | 1.2% | 1.3% |
| M50 | Cervical disc disorders | 1.9% | 0.8% | 1.4% | 1.8% |
| M43 | Other deforming dorsopathies | 1.9% | 1.1% | 1.1% | 1.2% |
| F43 | Reaction to severe stress, and adjustment disorders | < 1.2% | 1.9% | 0.9% | 0.7% |
| M16 | Coxarthrosis [arthrosis of hip] | < 1.2% | 2.1% | < 0.9% | 0.7% |
| F29 | Unspecified nonorganic psychosis | < 1.2% | < 0.8% | 1.4% | 1.5% |
| E66 | Obesity | < 1.2% | 1.0% | 1.4% | 1.0% |
| C50 | Malignant neoplasm of breast | < 1.2% | 1.8% | < 0.9% | 1.0% |
| G35 | Multiple sclerosis | < 1.2% | 1.0% | 1.1% | 0.9% |
| I63 | Cerebral infarction | 1.7% | < 0.8% | < 0.9% | 0.9% |

*Causes of disability pensions for patients younger than 65 at index were assessed. In these 2 964 (54%) patients per subgroup, disability pensions were observed in 417 (14.1%), 621 (21%), 570 (19.2%) and 678 (22.9%) of non-severe asthma and infrequent exacerbations, non-severe asthma and frequent exacerbations, severe asthma and infrequent exacerbations, and severe asthma and frequent exacerbations respectively. For example, in the severe asthma with frequent exacerbations group, 12.8% of all patients with disability pensions (N=87 of 678) had a disability pension due to asthma. Note that some patients have multiple disability pensions with different causes during the follow-up period.*

Supplementary Table 6. Comorbidities of unique ICD-10 codes on a three-character level, with a prevalence of 5% or more in any subgroup, based on data from 5 years preceding the index. Heat map proportions stratified by respiratory, CVD, metabolic, dental, musculoskeletal, and other diagnoses (blue – low prevalence; pale colouring – intermediate prevalence; red – high prevalence).

|  |  | **Non-severe asthma** | | **Severe asthma** | |  |  | **Fold difference** | |
| --- | --- | --- | --- | --- | --- | --- | --- | --- | --- |
|  | **Diagnosis** | **Infrequent exacerbations (n=5,525)** | **Frequent exacerbations (n=5,525)** | **Infrequent exacerbations (n=5,525)** | **Frequent exacerbations (n=5,525)** | **p-value all** | **Significant post-hoc tests** |  |  |
|  |  |  |  |  |  |  |  | **NSFE v NSIE** | **SFE v NSIE** |
| Respiratory | J20 - Acute bronchitis | 13.0 | 27.0 | 19.0 | 35.0 | <0.001 | All | 2.1 | 2.6 |
|  | J06 - Acute upper respiratory infections of multiple and unspecified sites | 17.0 | 28.0 | 22.0 | 32.0 | <0.001 | All | 1.6 | 1.8 |
|  | J12-J18 - Pneumonia | 6.6 | 19.0 | 12.0 | 29.0 | <0.001 | All | 2.9 | 4.5 |
|  | J01 - Acute sinusitis | 12.0 | 20.0 | 15.0 | 26.0 | <0.001 | All | 1.7 | 2.2 |
|  | J32 - Chronic sinusitis | 2.7 | 7.6 | 4.5 | 11.0 | <0.001 | All | 2.8 | 4.1 |
|  | J30 - Vasomotor and allergic rhinitis | 4.1 | 6.6 | 6.0 | 7.8 | <0.001 | NSFE/NSIE SIE/NSIE SFE/NSIE SFE/NSFE SFE/SIE | 1.6 | 1.9 |
|  | J33 - Nasal polyp | 1.7 | 6.4 | 1.9 | 6.2 | <0.001 | NSFE/NSIE SFE/NSIE SIE/NSFE SFE/SIE | 3.8 | 3.7 |
|  | J31 - Chronic rhinitis, nasopharyngitis and pharyngitis | 2.2 | 3.5 | 3.6 | 6.1 | <0.001 | NSFE/NSIE SIE/NSIE SFE/NSIE SFE/NSFE SFE/SIE | 1.6 | 2.8 |
|  | J47 - Bronchiectasis | 0.5 | 1.4 | 1.8 | 6.1 | <0.001 | NSFE/NSIE SIE/NSIE SFE/NSIE SFE/NSFE SFE/SIE | 2.6 | 11.6 |
|  | J22 - Unspecified acute lower respiratory infection | 1.5 | 3.6 | 2.6 | 5.9 | <0.001 | All | 2.4 | 4.0 |
| CVD | I10 - Essential (primary) hypertension | 31.0 | 36.0 | 33.0 | 38.0 | <0.001 | All | 1.2 | 1.2 |
|  | I48 - Atrial fibrillation and flutter | 8.9 | 11.0 | 9.4 | 13.0 | <0.001 | NSFE/NSIE SFE/NSIE SIE/NSFE SFE/NSFE SFE/SIE | 1.3 | 1.4 |
|  | I25 - Chronic ischaemic heart disease | 7.8 | 9.5 | 8.6 | 11.0 | <0.001 | NSFE/NSIE SFE/NSIE SFE/NSFE SFE/SIE | 1.2 | 1.4 |
|  | I50 - Heart failure | 4.0 | 7.6 | 6.2 | 10.0 | <0.001 | All | 1.9 | 2.5 |
|  | I49 - Other cardiac arrhythmias | 4.9 | 5.9 | 5.6 | 6.3 | 0.015 | NSFE/NSIE SFE/NSIE | 1.2 | 1.3 |
| Metabolic | E11 - Non-insulin-dependent diabetes mellitus | 12.0 | 14.0 | 13.0 | 16.0 | <0.001 | NSFE/NSIE SIE/NSIE SFE/NSIE SFE/NSFE SFE/SIE | 1.1 | 1.3 |
|  | E78 - Disorders of lipoprotein metabolism and other lipidaemias | 12.0 | 14.0 | 12.0 | 13.0 | 0.015 | NSFE/NSIE SIE/NSFE | 1.1 | 1.1 |
|  | E66 - Obesity | 4.0 | 7.2 | 5.8 | 9.0 | <0.001 | All | 1.8 | 2.3 |
|  | E03 - Other hypothyroidism | 5.9 | 7.7 | 6.4 | 8.3 | <0.001 | NSFE/NSIE SFE/NSIE SIE/NSFE SFE/SIE | 1.3 | 1.4 |
| Dental | K02 - Dental caries | 30.0 | 32.0 | 29.0 | 33.0 | <0.001 | NSFE/NSIE SFE/NSIE SIE/NSFE SFE/SIE | 1.1 | 1.1 |
|  | K03 - Other diseases of hard tissues of teeth | 14.0 | 14.0 | 15.0 | 16.0 | 0.007 | SFE/NSIE SFE/NSFE SFE/SIE | 1.0 | 1.2 |
|  | K04 - Diseases of pulp and periapical tissues | 13.0 | 15.0 | 14.0 | 16.0 | <0.001 | NSFE/NSIE SFE/NSIE SIE/NSFE SFE/SIE | 1.2 | 1.2 |
|  | K05 - Gingivitis and periodontal diseases | 10.0 | 10.0 | 9.4 | 10.0 | 0.4 |  | 1.0 | 1.0 |
| Musculoskeletal | M54 - Dorsalgia | 20.0 | 25.0 | 20.0 | 27.0 | <0.001 | NSFE/NSIE SFE/NSIE SIE/NSFE SFE/NSFE SFE/SIE | 1.3 | 1.4 |
|  | M79 - Other soft tissue disorders, not elsewhere classified | 13.0 | 19.0 | 15.0 | 20.0 | <0.001 | NSFE/NSIE SIE/NSIE SFE/NSIE SIE/NSFE SFE/SIE | 1.4 | 1.5 |
|  | M17 - Gonarthrosis [arthrosis of knee] | 12.0 | 15.0 | 13.0 | 15.0 | <0.001 | NSFE/NSIE SIE/NSIE SFE/NSIE SIE/NSFE SFE/SIE | 1.3 | 1.3 |
|  | M75 - Shoulder lesions | 9.9 | 13.0 | 9.9 | 12.0 | <0.001 | NSFE/NSIE SFE/NSIE SIE/NSFE SFE/SIE | 1.3 | 1.2 |
|  | M25 - Other joint disorders, not elsewhere classified | 7.4 | 10.0 | 8.2 | 11.0 | <0.001 | NSFE/NSIE SFE/NSIE SIE/NSFE SFE/SIE | 1.4 | 1.4 |
|  | M70 - Soft tissue disorders related to use, overuse and pressure | 6.0 | 7.6 | 5.9 | 8.9 | <0.001 | NSFE/NSIE SFE/NSIE SIE/NSFE SFE/NSFE SFE/SIE | 1.3 | 1.5 |
|  | M51 - Other intervertebral disc disorders | 4.3 | 6.7 | 4.9 | 6.7 | <0.001 | NSFE/NSIE SFE/NSIE SIE/NSFE SFE/SIE | 1.5 | 1.5 |
|  | M53 - Other dorsopathies, not elsewhere classified | 4.6 | 5.6 | 4.6 | 6.1 | <0.001 | NSFE/NSIE SFE/NSIE SIE/NSFE SFE/SIE | 1.2 | 1.3 |
|  | M48 - Other spondylopathies | 3.5 | 4.6 | 3.7 | 6.0 | <0.001 | NSFE/NSIE SFE/NSIE SIE/NSFE SFE/NSFE SFE/SIE | 1.3 | 1.7 |
|  | M16 - Coxarthrosis [arthrosis of hip] | 4.6 | 5.6 | 4.9 | 5.9 | 0.008 | NSFE/NSIE SFE/NSIE SFE/SIE | 1.2 | 1.3 |
|  | M15 - Polyarthrosis | 3.3 | 5.4 | 3.7 | 5.2 | <0.001 | NSFE/NSIE SFE/NSIE SIE/NSFE SFE/SIE | 1.6 | 1.6 |
|  | M47 - Spondylosis | 3.6 | 4.1 | 3.4 | 5.2 | <0.001 | SFE/NSIE SFE/NSFE SFE/SIE | 1.1 | 1.5 |
|  | M19 - Other arthrosis | 3.7 | 5.1 | 4.6 | 5.0 | 0.002 | NSFE/NSIE SIE/NSIE SFE/NSIE | 1.4 | 1.3 |
| Other | G47 - Sleep disorders | 6.0 | 9.0 | 8.3 | 12.0 | <0.001 | NSFE/NSIE SIE/NSIE SFE/NSIE SFE/NSFE SFE/SIE | 1.5 | 1.9 |
|  | H25 - Senile cataract | 7.6 | 9.4 | 9.0 | 11.0 | <0.001 | NSFE/NSIE SIE/NSIE SFE/NSIE SFE/NSFE SFE/SIE | 1.2 | 1.4 |
|  | N30 - Cystitis | 5.8 | 8.3 | 7.0 | 9.2 | <0.001 | NSFE/NSIE SIE/NSIE SFE/NSIE SIE/NSFE SFE/SIE | 1.4 | 1.6 |
|  | F32-F33 - Depression | 5.8 | 9.0 | 7.7 | 8.9 | <0.001 | NSFE/NSIE SIE/NSIE SFE/NSIE SIE/NSFE SFE/SIE | 1.5 | 1.5 |
|  | N39 - Other disorders of urinary system | 5.7 | 7.7 | 6.4 | 8.8 | <0.001 | NSFE/NSIE SFE/NSIE SIE/NSFE SFE/NSFE SFE/SIE | 1.4 | 1.5 |
|  | K21 - Gastro-oesophageal reflux disease | 4.5 | 5.2 | 5.8 | 7.5 | <0.001 | SIE/NSIE SFE/NSIE SFE/NSFE SFE/SIE | 1.2 | 1.6 |
|  | H10 - Conjunctivitis | 5.4 | 7.0 | 6.0 | 7.4 | <0.001 | NSFE/NSIE SFE/NSIE SIE/NSFE SFE/SIE | 1.3 | 1.4 |
|  | K57 - Diverticular disease of intestine | 5.5 | 7.5 | 6.1 | 7.4 | <0.001 | NSFE/NSIE SFE/NSIE SIE/NSFE SFE/SIE | 1.4 | 1.3 |
|  | A09 - Diarrhoea and gastroenteritis of presumed infectious origin | 3.1 | 5.6 | 4.2 | 7.2 | <0.001 | All | 1.8 | 2.3 |
|  | H90 - Conductive and sensorineural hearing loss | 5.7 | 7.0 | 6.0 | 7.1 | 0.005 | NSFE/NSIE SFE/NSIE SIE/NSFE SFE/SIE | 1.2 | 1.2 |
|  | A46 - Erysipelas | 3.2 | 5.6 | 4.7 | 6.8 | <0.001 | NSFE/NSIE SIE/NSIE SFE/NSIE SFE/NSFE SFE/SIE | 1.7 | 2.1 |
|  | L30 - Other dermatitis | 4.9 | 7.4 | 5.0 | 6.8 | <0.001 | NSFE/NSIE SFE/NSIE SIE/NSFE SFE/SIE | 1.5 | 1.4 |
|  | K08 - Other disorders of teeth and supporting structures | 4.2 | 5.4 | 4.5 | 6.0 | <0.001 | NSFE/NSIE SFE/NSIE SIE/NSFE SFE/SIE | 1.3 | 1.4 |
|  | G44 - Other headache syndromes | 3.8 | 4.8 | 3.8 | 5.5 | <0.001 | NSFE/NSIE SFE/NSIE SIE/NSFE SFE/SIE | 1.2 | 1.4 |
|  | K59 - Other functional intestinal disorders | 3.3 | 4.2 | 3.6 | 5.4 | <0.001 | NSFE/NSIE SFE/NSIE SFE/NSFE SFE/SIE | 1.3 | 1.7 |
|  | F41 - Other anxiety disorders | 3.1 | 4.1 | 3.8 | 5.0 | <0.001 | NSFE/NSIE SIE/NSIE SFE/NSIE SFE/NSFE SFE/SIE | 1.3 | 1.6 |

NSFE- non-severe asthma and frequent exacerbations; NSIE - non-severe asthma and infrequent exacerbations; SFE - severe asthma and frequent exacerbations; SIE - severe asthma and infrequent exacerbations.
