# Supplementary figures and images for: Burden of asthma by severity and exacerbation frequency among adult patients naive to biologic asthma therapy: A Finnish cohort study
Source: J Allergy Clin Immunol Glob. 2025 Mar 14;4(2):100453. doi: 10.1016/j.jacig.2025.100453 (PMC12018094; doi:10.1016/j.jacig.2025.100453)

# Mortality

Annual mortality rate per patient year

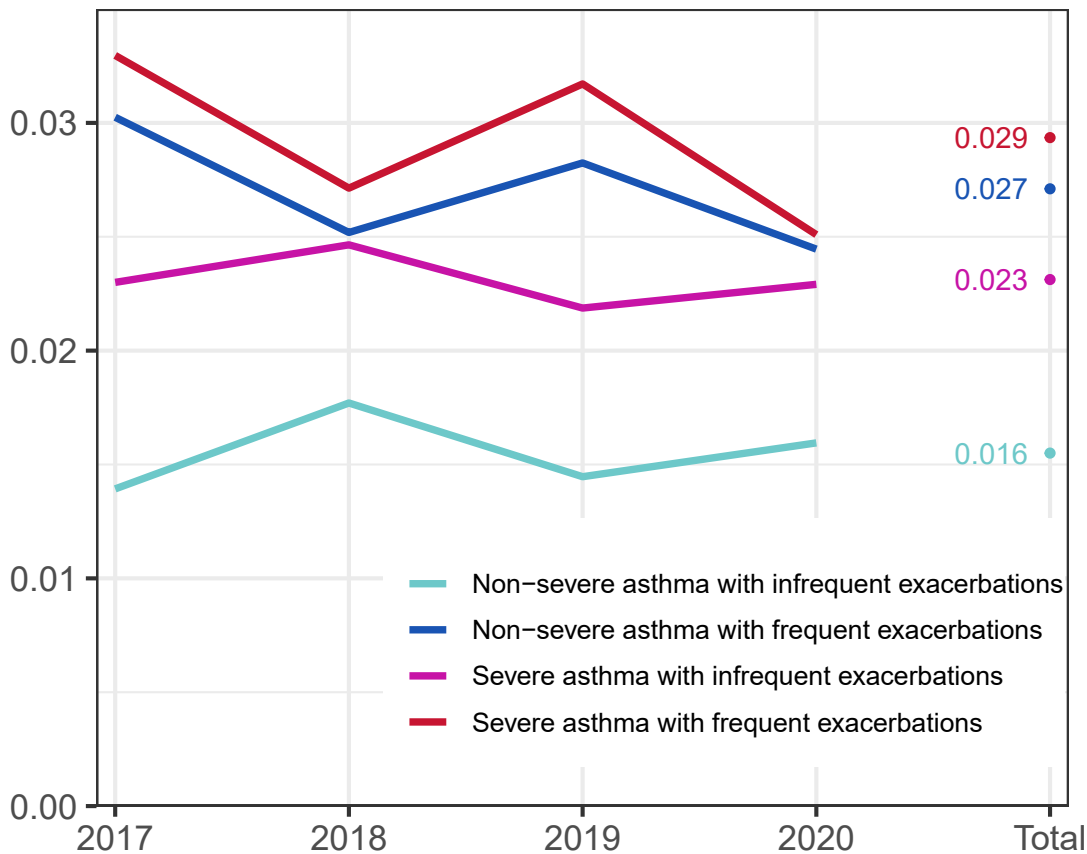

Supplement: Supplementary Figure 1 [file mmc2.pdf]
