# Supplementary material for: Burden of asthma by severity and exacerbation frequency among adult patients naive to biologic asthma therapy: A Finnish cohort study
Source: J Allergy Clin Immunol Glob. 2025 Mar 14;4(2):100453. doi: 10.1016/j.jacig.2025.100453 (PMC12018094; doi:10.1016/j.jacig.2025.100453)
Supplement: Supplementary Figure 2 [file mmc3.pdf]

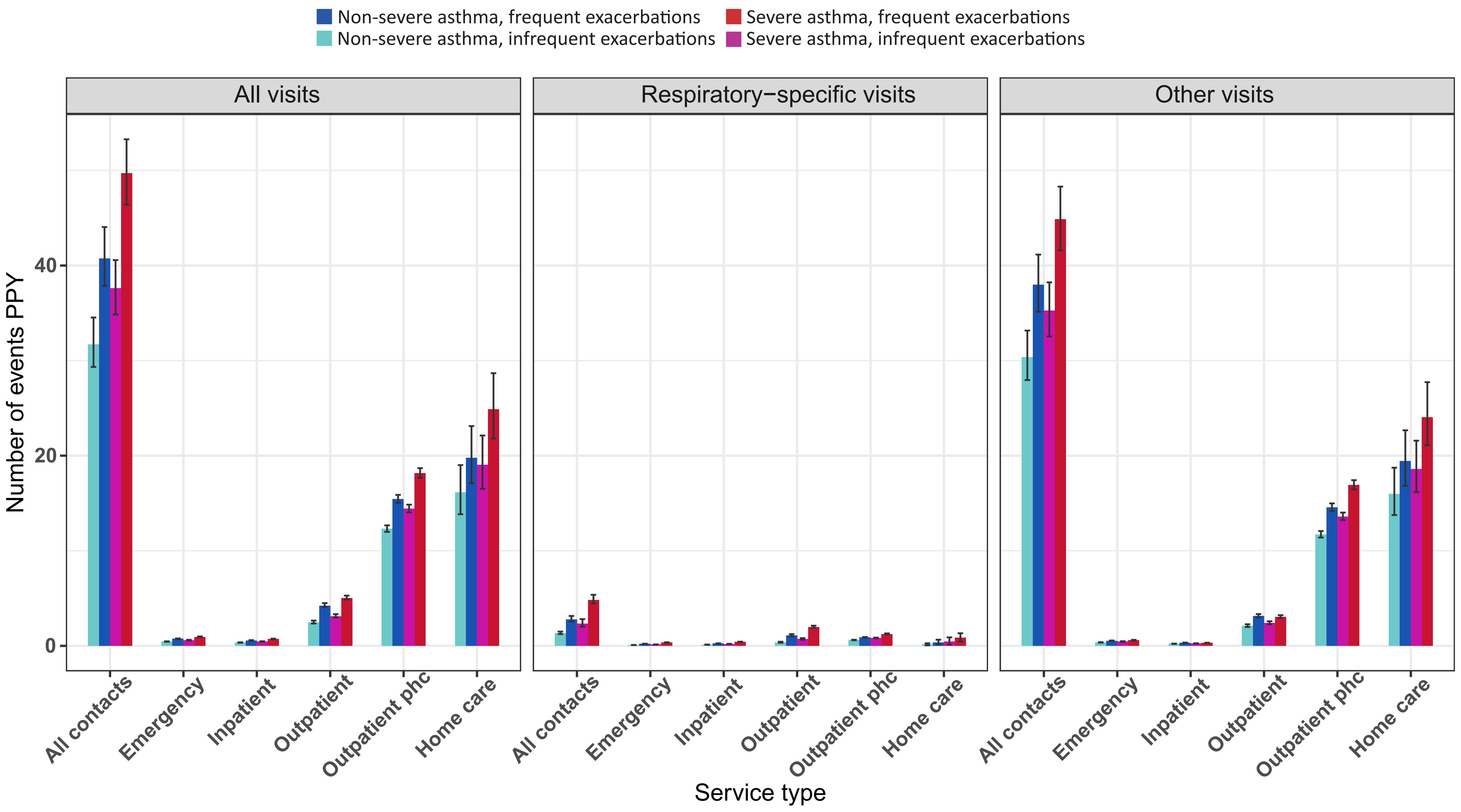

Non-severe asthma, frequent exacerbations
Non-severe asthma, infrequent exacerbations
Severe asthma, frequent exacerbations
Severe asthma, infrequent exacerbations
All visits
Respiratory−specific visits
Other visits
Number of events PPY
0
20
40
All contacts
Emergency
Inpatient
Outpatient
Outpatient phc
Home care
Service type
